# Severe gastrointestinal symptoms during first year after laparoscopic cholecystectomy

**DOI:** 10.12669/pjms.41.1.10125

**Published:** 2025-01

**Authors:** Saleh Abdulrahman Almulhim, Mounther Mohammed AlNaim, Abdul Qadeer Memon, Abdulrhman Khalid Aldrweesh, Omar Ahmed Aldamigh, Faisal Abdulaziz Alhadi, Shahd Salah Albooshal, Abdullah Ahmed Alabdrabulridha, Ahmed Hassan Kamal, Abdulrahman Saleh Al-Mulhim

**Affiliations:** 1 Saleh Abdulrahman Almulhim, MBBS Family Medicine Physician, National Guard Hospital AlAhsa, Saudi Arabia; 2 Mounther Mohammed AlNaim, MBBS, SBFM, ABFM Associate Consultant in Family Medicine, National Guard Hospital AlAhsa, Saudi Arabia; 3 Abdul Qadeer Memon, FCPS Assistant Professor in Surgery, King Faisal University College of Medicine, Riyadh, Saudi Arabia; 4 Abdulrhman Khalid Aldrweesh, MBBS King Faisal University College of Medicine, Riyadh, Saudi Arabia; 5 Omar Ahmed Aldamigh, MBBS King Faisal University College of Medicine, Riyadh, Saudi Arabia; 6 Faisal Abdulaziz Alhadi, MBBS King Faisal University College of Medicine, Riyadh, Saudi Arabia; 7 Shahd Salah Albooshal, MBBS King Faisal University College of Medicine, Riyadh, Saudi Arabia; 8 Abdullah Ahmed Alabdrabulridha, MBBS King Faisal University College of Medicine, Riyadh, Saudi Arabia; 9 Ahmed Hassan Kamal Assistant Professor in Orthopedics, King Faisal University College of Medicine, Riyadh, Saudi Arabia; 10 Abdulrahman Saleh Al-Mulhim, FRCSI, FICS, FACS Professor in Surgery, King Faisal University College of Medicine, Riyadh, Saudi Arabia

**Keywords:** Cholelithiasis, Cholecystitis, Laparoscopic cholecystectomy, Gastrointestinal symptoms

## Abstract

**Background & Objective:**

Many patients after cholecystectomy, develop gastrointestinal (GI) symptoms and are worried. The objective of this study was to find out the risk factors for severe GI symptoms following laparoscopic cholecystectomy (LC) during first year of follow-up.

**Methods:**

It is a multi-center prospective observational study. In this study, patients who underwent LC for gallstones as elective or emergency at different hospitals of Al-Ahsa region of the Kingdom of Saudi Arabia from January 2020 to December 2021were evaluated for the development of severe GI symptoms postoperatively. They were followed-up for one year after the surgery.

**Results:**

One thousand and two patients who underwent LC, 139(13.87%) developed severe GI symptoms after LC. The patients affected were mostly females 103(74.1%). After LC, the GI symptoms observed were increased frequency of defecation 113(81.3%), loose stools 102 (73.4%), urgency 63(45.3%) and bloating 60 (43.16%) patients. Forty-seven (33.8%) patients had two GI symptoms at a time, followed by 36 (25.9%) who had three. The median time of the start of GI symptoms was two weeks after surgery and the symptoms ended at 12 weeks after surgery. Forty-five (32.4%) patients had less than three-weeks’ duration, followed by 35(25%) having less than 15 weeks. A BMI more than 40 showed a significant association. A higher frequency of cholecystitis attacks and a delayed onset of GI symptoms showed a significant severity of GI symptoms, resulting in delayed resolution. The bowel symptoms were more prominent in the emergency group. Age and gender had no significance.

**Conclusion:**

Early laparoscopic cholecystectomy of patients with cholelithiasis who have a smaller number of attacks of cholecystitis have less frequency of severe GI symptoms after the surgery.

## INTRODUCTION

Laparoscopic cholecystectomy (LC) has been widely accepted for many decades as the preferred and most effective treatment for symptomatic gallstone disease, which affects approximately 22% of adults.^1^,^2^ LC can be carried out either on an elective basis for gallstones or on an emergency basis when admitted to the hospital.^3^ Numerous studies and evidence advocate the adoption of laparoscopic surgery as a standard approach, mainly due to its significantly reduced risk of complications and mortality when compared to open cholecystectomy.^1^

Many patients may present with gastrointestinal (GI) symptoms after LC ranging from mild to severe, which include abdominal pain, flatulence, dyspepsia, or altered bowel habits.^4^,^5^ Out of all the GI symptoms experienced after cholecystectomy, post-laparoscopic cholecystectomy diarrhea (PLCD) is particularly serious and frequently reported, persisting for a prolonged duration following the surgical procedure.^6^ A study has observed that diarrhea after cholecystectomy can continue for up to four years postoperatively.^7^ The prevalence of PLCD varies across studies, with reported rates ranging from 0.9% to 35.6%.^5^

The precise mechanism underlying postoperative bowel changes subsequent to LC remains incompletely understood. Nonetheless, several theories have been proposed. One theory suggests that PLCD arises as a consequence of the loss of a primary bile acid reservoir, leading to prolonged interaction between bile acids and intestinal mucus, ultimately resulting in bile acid malabsorption and diarrhea. Another theory suggests that the removal of the gallbladder leads to a shortened gut transit time.^1^,^5^ The associated factors and predictors of PLCD have not been extensively studied or fully realized, nor has the optimal timing for performing LC been determined. This study aimed to identify predisposing risk factors for alterations in bowel habits following LC.

## METHODS

This is a multi-center prospective observational study to find out the risk factors and predictors of GI symptoms following LC. All the patients who underwent LC for gallstones as elective or emergency at different hospitals of Al-Ahsa region of the Kingdom of Saudi Arabia from January 2020 to December 2021 were considered for the study. Out of which, 1002 patients completed the follow-up for one year were included. The patients who had open cholecystectomy for gallstones, LC for other than gallstones, the patients having choledocholithiasis or who did not complete the one-year follow-up after LC were excluded.

### Ethical considerations:

Ethical approval was obtained from the King Faisal University Ref. No.: 2020-12-48, dated December 27, 2020. Patients’ confidentiality and privacy were ensured throughout the study and informed consent was obtained from participants.

### Data Collection:

Patients’ demographics, medical history, surgical details, and GI symptoms were recorded. Variables of interest included age, gender, BMI, comorbidities (diabetes, hypertension, thyroid disease), number of cholecystitis attacks before surgery, waiting time (from less than two months to more than 12 months) from the first attack of cholecystitis to surgery, duration of symptoms before relief, onset and duration of GI symptoms after surgery and type of cholecystectomy (elective or emergency).

The severity of gastrointestinal symptoms was evaluated through patient interviews conducted during the follow-up period. Gastrointestinal symptoms were categorized as “none”, “weak” and “severe” with corresponding numerical values of Zero, one and two respectively. This categorization method was derived from the research conducted by Kim GH et al.^8^

### Statistical analysis:

The collected data were analyzed using IBM SPSS version 25 (IBM Corp., Armonk, NY). Data exploration indicated that the data were not normally distributed. As a result, frequencies were employed to describe categorical variables for descriptive statistics, while medians and interquartile ranges (IQRs) were utilized to report continuous variables. The associations between demographic characteristics (predictors) and the duration of severe symptoms were investigated using univariate and multivariable ordinal logistic regression analysis. Significant results were defined as a P value with a 95% confidence interval of less than 0.05.

## RESULTS

### Demographics:

One hundred-thirty-nine patients out of 1002, who had LC, experienced severe GI symptoms. Among them, 139(13.78%) patients experienced persistent GI symptoms after the surgery. Out of these, 103(74.1%) were females and 36(25.9%) were males. The patients affected were mostly females 103(74.1%). Their median age was 38 and (the range was 21-67) years. Twenty-seven (19.4%) had diabetes, 27(19.4%) had hypertension, 4(2.9%) had hyperthyroidism and 1(0.7%) had hypothyroidism. Eighteen (12.9%) patients had their BMI less than 30, 80(57.6%) had between 30 to 40 and 41(29.5%) had more than 40. Fifty-four (38.8%) patients had more than ten attacks of cholecystitis prior to surgery, 48(34.5%) had the surgery done in less than two months following the first attack of cholecystitis and 73(52.5%) had follow-up of three to 12 months (Table-I).

**Table-I:** Demographic data of the patients (n=139).

Variable	Finding n(%)
***Age***	
Median	38.00
Minimum	21.00
Maximum	67.00
***Gender***	
Females	103(74.1)
Males	36(25.9)
***BMI***	
<30	18(12.9)
30-40	80(57.6)
>40	41(29.5)
***No. of attacks of cholecystitis from 1^st^ attack to surgery***	
1-3 times	44(31.7)
<10 times	41(29.5)
>10 times	54(38.8)
***Time duration from 1^st^ attack of cholecystitis to surgery***	
<2 months	48(34.5)
2-12 months	45(32.4)
>12 months	46(33.1)
***Follow-up***	
<3 months	37(26.6)
3-12 months	73(52.5)
>12 months	29(20.9)
***Mode of surgery***	
Elective	86(61.9)
Emergency	53(38.1)
***Diabetes mellitus***	
No	112(80.6)
Yes	27(19.4)
***Hypertension***	
No	112(80.6)
Yes	27(19.4)
***Thyroid function***	
Hyperthyroidism	4(2.9)
Hypothyroidism	1(0.7)
***Start of GI symptoms (Weeks)***	
Median	2.00
Minimum	1.00
Maximum	3.00
***End of GI symptoms (Weeks)***	
Median	12.00
Minimum	4.00
Maximum	40.00

### GI symptoms:

After LC, the GI symptoms observed were loose stools in 102 (73.4%), increased frequency of defecation in 113(81.3%), urgency in 63(45.3%) and bloating in 60 (43.16%) patients. Twenty-nine (20.9%) patients had one, 47 (33.8%) had two, 36(25.9%) had three, followed by 27(19.4%) who had four GI symptoms at a time. The median time for the start of the GI symptoms was two weeks after surgery (range 1-3) and for the end of the symptoms, it was 12 (range 4-40) weeks. Forty-five (32.4%) patients had less than three, 28(20.1%) had less than six, 35(25.2%) had less than 15 and 31(22.3%) patients had more than fifteen weeks duration of severe symptoms (Table-II).

**Table-II:** Findings of the GI symptoms (n=139).

Symptom	Frequency (%)
***Loose stools***	
No	37(26.6)
Yes	102(73.4)
***Increased frequency of defecation***	
No	26(18.7)
Yes	113(81.3)
***Urgency***	
No	76(54.7)
Yes	63(45.3)
***Bloating***	
No	79(56.8)
Yes	60(43.2)
***Number of symptoms***	
One	29(20.9)
Two	47(33.8)
Three	36(25.9)
Four	27(19.4)
***Duration of severe Symptoms***	45(32.4)
=<3 weeks	
=<6 weeks	28(20.1)
=<15 weeks	35(25.2)
>15 weeks	31(22.3)

Univariate analysis revealed that a BMI of more than 40 (COR 74, 95% CI 21-296), a higher number of cholecystitis attacks before surgery (COR 4, 95% CI 2-10), longer waiting time from first attack to surgery (COR 15, 95% CI 6-36), a higher number of GI symptoms (COR 3,95%CI 1.2-8), longer duration of GI symptoms to subside (COR 1.6, 95% CI 1.4-1.9) and patients who had emergency cholecystectomy (COR 1.9, 95% CI 1.02-3.7) were significantly associated with increased odds of having more prolonged duration of severe symptoms. However, other predictors like higher age (COR 0.89, 95% CI 0.86-0.92) and later start of symptoms postoperatively (COR 0.11, 95 % CI 0.05-0.2) were significantly associated with shorter duration of severe GI symptoms. Other predictors (gender, DM, hypertension, thyroid disease) did not show a significant association (Table-III).

**Table-III:** Shows the impact of demographics on severity of GI symptoms using univariate and multivariable ordinal logistic regression.

Univariate analysis					Multivariable analysis				

Predictors	COR	COR 95% confidence interval		P-value	AOR		AOR 95% confidence interval		P-value

		lower	Higher				lower	Higher	
Age	0.891	0.86	0.921	<.001*	0.9615		0.91299	1.012	0.134
Gender									
F – M	0.724	0.362	1.45	0.36	1.0661		0.46913	2.445	0.879
BMI									
30- 40 – <30	2.85	1.04	8.74	0.05*	1.0446		0.33041	3.587	0.942
≥40 – <30	74.24	21.13	296.5	<.001*	12.5704		2.98551	57.602	<.001*
***No of attacks of cholecystitis before surgery***									
1 TO 3 – >10	34.99	13.51	96.7	<.001*	1.391		0.3514	5.47	0.635
<10 – >10	4.44	2	10.3	<.001*	1.785		0.3278	9.76	0.501
***1^st^ attack of cholecystitis to surgery***									
<2month – ≥12 month	15.01	6.41	36.85	<.001*	0.3804		0.07574	1.792	0.225
2 TO 12 – ≥12 month	2.64	1.22	5.84	0.015*	0.0768		0.00847	0.598	0.017*
***Emergency***									
Yes – No	1.95	1.02	3.74	0.043*	2.6931		0.89241	8.308	0.08
***Start of symptoms***									
	0.118	0.0576	0.227	<.001*	0.3182		0.15009	0.649	0.002*
***End of symptoms***									
	1.64	1.45	1.92	<.001*	1.605		1.3988	1.91	<.001*
***Total number of symptoms***									
2 – 1	1.15	0.508	2.61	0.742	2 – 1		0.527	0.1742	1.57
3 – 1	2.62	1.12	6.23	0.028*	3 – 1		0.909	0.2411	3.44
4 – 1	3.11	1.207	8.21	0.02*	4 – 1		0.44	0.0628	2.89
DM									
Yes – No	0.891	0.387	2.04	0.785	1.842		0.4852	7.5	0.378
***Hypertension***									
No – Yes	0.692	0.319	1.49	0.347	0.805		0.2371	2.75	0.726
***Thyroid***									
No – Yes	0.259	0.0501	1.14	0.079	8.334		0.416	174.85	0.162

* Significant, COR= Crude Odd Ratio, AOR= Adjusted Odd Ratio. (N=139).

When all predictors were added to the ordinal logistic regression model, multivariate analysis revealed that BMI > 40 (AOR 12, 95% CI 2.9-57) and longer duration of symptoms to subside (AOR 1.6 95% CI 1.3-1.9) were significantly associated with higher odds of having a longer duration of severe symptoms. Nevertheless, an increased number of cholecystitis attacks (AOR 0.07, 95% CI 0.008-0.5) and later start of GI symptoms (AOR 0.3, 95% CI 0.15-0.64) were associated with significantly lower duration of severe GI symptoms. All other predictors did not show significant associations (Table-III).

## DISCUSSION

In this study, out of 1002 patients having LC, 139(13.78%) suffered from persisting GI symptoms postoperatively, with women having larger rate 103(74.1%). A study by Fisher M et al. has identified numerous risk factors that predispose patients to post-cholecystectomy diarrhea which include younger age, male sex, and higher BMI^2^. On multivariate analysis of this study, higher BMI (more than 40) showed a significant and independent association with risks of acquiring longer periods of GI symptoms postoperatively (P=0.001). Obesity itself is cause of many GI related symptoms.^9^ In this study, the obese patients who developed postoperative GI symptoms were not having such symptoms preoperatively. The PLCD arises as a consequence of the loss of a primary bile acid reservoir, leading to prolonged interaction between bile acids and intestinal mucus, ultimately resulting in bile acid malabsorption and diarrhea.^5^

Age (P=0.134) and gender (P=0.879) had no significant association with the development of GI symptoms after the surgery (Table-III). Several previous studies have advocated early LC as a more suitable option for the treatment of biliary disease with a fewer incidence of clinical complications that occur due to postponing surgical intervention.^10^-^12^ LC has shown to have high rates of success and patient satisfaction. However, it is also evident that some patients do not achieve full symptom-relief after the surgery and continue to experience a few non-pain symptoms, with diarrhea being the most distressing among them.^2^

In the multivariate analysis of this study, longer duration of symptoms before LC was significantly linked to extended periods of GI symptoms after surgery (P=0.001). However, a higher frequency of cholecystitis attacks and a delayed onset of GI symptoms showed a significant increase in the severity of GI symptoms, resulting in delayed resolution. A study conducted by Arora D et al. has shown that the occurrence of prior cholecystitis attacks has been repeatedly recognized as a risk factor for the symptoms following LC.^13^ While we acknowledged this factor in our study, it was found that the symptoms associated with it tended to occur less frequently and persist for shorter durations because we were targeting the severe symptoms only.

The outcomes after cholecystectomy have been explored in various studies. Middelfart H V et al. found that outcomes were independent of whether the operation was elective or following acute cholecystitis.^14^ Nevertheless, our findings on univariate analysis suggest that bowel symptoms were more prominent in the emergency group (P=0.043) (Table-III), which may be attributed to differences in patient selection or initial management. Because some patients with acute cholecystitis are being operated within 48-72 hours after initial conservative treatment and stabilization, while others are treated conservatively followed by interval elective cholecystectomy if they get relief from the symptoms. The most prominent GI symptom in this study was found to be increased frequency of defecation. A study conducted by Jasim H et al. aimed to assess the effect of cholecystectomy on bowel habits and has shown an increase in bowel motion frequency with the highest trend from two weeks to three months after surgery.^4^ This may be due to the patients might regain their suppressed appetite after cholecystectomy, which increases their dietary intake and leads to the frequent bowel movements that might be perceived as diarrhea.

The findings of the current study align with what was demonstrated by Del Grande LM et al., showing no significant correlation with diabetes (P=0.785), hypertension (P=0.347) and thyroid disease (P=0.079) with diarrhea and other gastrointestinal symptoms (Table-III).^5^ Few studies have suggested the involvement of various personality traits in the interpretation of postoperative symptoms and sensations and how psychological vulnerability might negatively impact patients’ experience after the surgery putting them at risk of developing persistent GI symptoms.^6^,^15^ We have not tested that hypothesis in this study.

Thunnissen FM et al. evaluated two prospective studies, in which he observed that the patients who had biliary colic and underwent cholecystectomy; after six months’ follow-up, most of the abdominal symptoms subsided significantly but the abdominal pain remained persistent. The persistent symptoms were flatulence and restricted eating in 17.8% and 14.5% respectively. They also observed some new-onset symptoms like frequent bowel movements, bowel urgency and diarrhea.^16^ Cholecystecomy is also found to be associated with irritable bowel syndrome (IBS)-like symptoms with diarrhea in patients in the UK. It was contrarily found that the patients who had cholecystectomy due to biliary colic and were regularly using NSAIDs, had less frequent IBS. However, the relationship between the IBS and use of NSAIDs was unclear.^17^,^18^ The factors which may determine the persistence of symptoms like diarrhea after cholecystectomy include preoperative dyspepsia, functional disorders, atypical pain locations, longer duration of symptoms, and poor psychological or physical health.^19^ Bile acid diarrhea is one of the causes of persistence of post-cholecystecomy diarrhea. It occurs due to disruption of the enterohepatic circulation, causing hepatic overproduction of bile acids.^20^ The findings of this study are synchronous with the above studies in relation with the higher BMI, use of NSAIDs for biliary colic and post-LC GI symptoms. The significance of this study lies in that the patients with cholelithiasis may be operated at the earliest available elective list without giving it time for complications to develop. More episodes of complications, especially cholecystitis, will lead to persistent GI complications even after cholecystectomy.

### Limitations:

First, there is a lack of a control group. Secondly, it did not focus on assessing the quality of life (QoL) differences between individuals who develop GI symptoms postoperatively and those who experience good relief of symptom. Despite these limitations, we were able to evaluate the development of persistent symptoms in a large number of patients for more than 12 months after hospital discharge, providing a more accurate reflection of the prevalence of persistent GI symptoms following surgery in the region.

## CONCLUSION

Laparoscopic cholecystectomy of the patients with cholelithiasis at the earliest possible time, who have less frequency of cholecystitis attacks and are not obese, have less frequency of severe GI symptoms after the surgery.

### Recommendations:

In future research, it is necessary to focus on assessing QoL through long-term follow-up. Additionally, it is important to identify the precise mechanisms that contribute to the development of persistent GI symptoms after surgery.

### Authors’ contribution:

**SAA, MMA, AAK**, **AOA, FAA**, **SSA and AAA:** Sorted out the data and were involved in manuscript preparation.

**AQM:** Analyzed, edited and reviewed the manuscript.

**AHK:** Analyzed and reviewed the manuscript.

**ASA:** Written, Analyzed, edited, reviewed, and finally approved the manuscript. He is also responsible and accountable for the accuracy and integrity of the work.

All authors have read & approved the manuscript.
